# Benchmarking the medication efficiency and technological progress of diabetes drugs

**DOI:** 10.3389/fpubh.2024.1396832

**Published:** 2024-11-08

**Authors:** Hongwei Zhang, Chen Wang, Ting Xu, Lin Liu, Xuyan Ban, Weijie Liu, Chenli Yan, Xiaodong Han

**Affiliations:** ^1^Department of Metabolic and Bariatric Surgery, Shanghai Sixth People’s Hospital Affiliated to Shanghai Jiao Tong University School of Medicine, Shanghai, China; ^2^Technological Economics and Management, School of Business Administration, Capital University of Economics and Business, Beijing, China

**Keywords:** diabetes, medication efficiency, technological progress, directional distance model, efficiency change, frontier, DEA

## Abstract

**Background:**

Diabetes poses a serious global challenge, given its increasing prevalence, detrimental effects on public health, and substantial economic burden. Since 1950s, tens of drugs have been approved by the United States (US) Food and Drug Administration (FDA). In the past decade, the medical community and regulatory agencies have moved away from the glucose-centric paradigm and increasingly call for a holistic approach to assess different treatments’ benefits and harms.

**Objective:**

This study aimed to assess the medication efficiency and technological progress of Type 2 Diabetes (T2D) drugs, by considering their physiological outcomes, including both benefits (i.e., glucose lowering and weight loss) and adverse effects (mortality), relative to dosing frequency.

**Methods:**

To derive medication efficiency, this study utilized data from the US FDA and prominent meta-analyses. Given that both the benefits and adverse effects of medications are multidimensional, this study employed a nonparametric frontier method, the data envelopment analysis (DEA) model, to integrate these factors into a measure of medication efficiency. Physiological outcomes could assume both positive and negative values. Adverse effects were regarded undesirable outputs. The DEA model was built under the framework of directional distance function and was able to handle negative and undesirable values which naturally arose in the case of T2D medications.

**Results:**

The paper presented a ranking of 20 T2D drugs in terms of medication efficiency. Three of them were able to attain the highest medication efficiency, all of which were in the GLP-1 class, including oral Semaglutide, subcutaneous Semaglutide and Dulaglutide. However, the other two GLP-1 drugs, Lixisenatide and Liraglutide, were less efficient. The average medication efficiency of drugs approved post-2010 was significantly higher than pre-2010 drugs. High dose frequency, low HbA1c reduction and insignificant weight loss were the main driving factors behind inefficiencies. Overall, medication efficiency provided an alternative perspective on treatment effectiveness other than conventional measures such as cost-effectiveness.

## 1 Introduction

Diabetes presents a severe global challenge, underscored by compelling quantitative figures that highlight its pervasive impact on health, economies, and quality of life. According to the International Diabetes Federation (IDF), approximately 537 million adults (20–79 years) were living with diabetes worldwide in 2021 and this number is projected to rise to 783 million by 2045 (1). Diabetes also imposes a substantial economic burden for countries around the world. For example, the American Diabetes Association (ADA) estimated the total cost of diagnosed diabetes in the United States (US) to be $412.9 billion in 2017, including direct medical costs and indirect costs such as productivity loss. Moreover, Diabetes is a major contributor to illness and death worldwide. It elevates the likelihood of several serious complications such as cardiovascular disease, strokes, kidney failure, vision loss, and amputations of the lower limbs. In 2021, diabetes was linked to approximately 6.7 million deaths globally, accounting for 11% of fatalities among people aged 20–79 (1).

**Figure 1:**
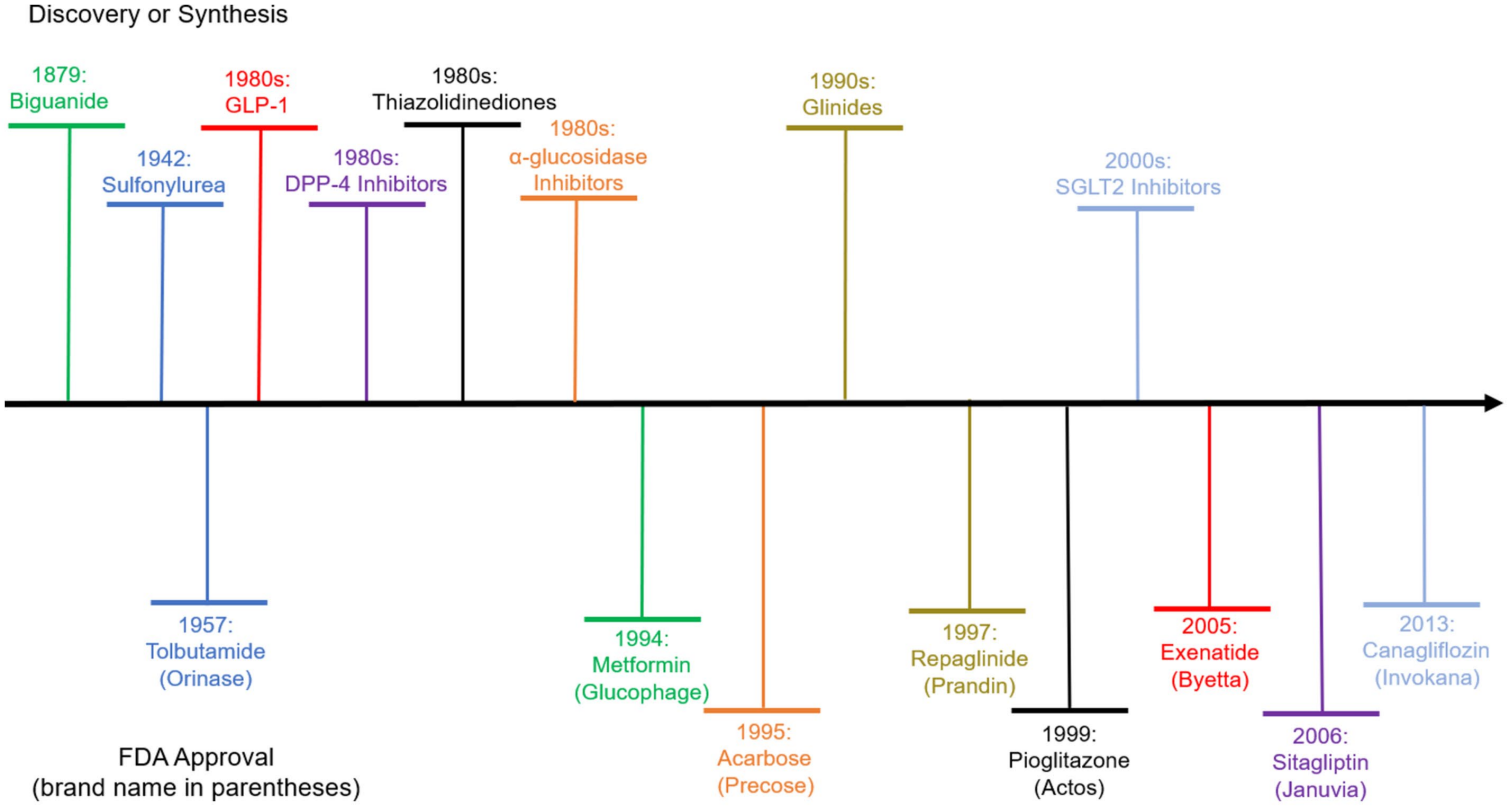
Evolution of diabetes drugs. The upper part above the horizontal axis shows the discovery of the chemical agent classes that the drugs belong to. The lower part shows the compound names and the corresponding brand names of the drugs of each class that is first approved by the US FDA.

Anti-diabetic drugs play a critical role against the disease (2). Figure 1 plots the evolution of Type 2 Diabetes (T2D) drugs, including the discovery of the compounds and the earliest approvals of the compounds with brand names by the US Food and Drug Administration (FDA). The first approval was granted to Tolbutamide under the brand name Orinase under the class of Sulfonylurea in 1957. A second class of drugs, the Biguanide, was approved in 1994 for the compound Metformin. Recent years see the approval of GLP-1 (e.g., Exenatide), DPP-4 inhibitors (e.g., Sitagliptin), and SGLT2 inhibitors (e.g., Canagliflozin).

The primary goal of anti-diabetic drugs is to regulate blood glucose levels (3). Effective control helps prevent hyperglycemia (high blood sugar) and reduce the risk of acute complications. Intensive blood glucose control, particularly with certain medications like insulin and sulfonylureas, can increase the risk of hypoglycemia, which can lead to dizziness, confusion, and in severe cases, unconsciousness. Some diabetes medications, such as certain types of insulin and sulfonylureas, may be associated with weight gain, which can exacerbate other risk factors for diabetes-related complications. Some individuals may experience gastrointestinal side effects, such as nausea or diarrhea, with certain diabetes medications. In some cases, there may be concerns about potential cardiovascular risks associated with specific classes of diabetes drugs. The cardiovascular safety of new medications is a key consideration during regulatory evaluations. Certain medications may pose a risk to kidney function, especially in individuals with pre-existing kidney disease.

This paper examines the *medication efficiency* of T2D drugs. Here, medication efficiency is defined as the by the balance between therapeutic benefits and side effects, in relation to the frequency of dosing (i.e., the number of doses taken per day or per week). It gauges the extent of health improvement achieved with each dose. According to this definition, if two drugs—A and B—produce identical therapeutic benefits and side effects, but Drug A requires daily dosing while Drug B is taken weekly, Drug B would be considered more efficient. It is important to distinguish medication efficiency from three other related yet distinct concepts, medication effectiveness, dose-effectiveness and cost-effectiveness. Medication effectiveness refers to how well a medication achieves its intended therapeutic effect. On the other hand, medication efficiency refers to how well a medication achieves its desired effect while using the least amount of dose. Dose-effectiveness refers to the relationship between the dose (amount or concentration) of a drug administered and the resulting effects it produces on the body. Cost-effectiveness emphasizes the cost per unit of health improvement.

Medication efficiency provides a unique and insightful perspective on the effectiveness of medications, offering advantages over other measures for two reasons. Firstly, it considers patient behavior and adherence by incorporating the dosing schedule into its definition. Medications that require fewer doses reduce the patient’s pill burden, thereby enhancing convenience and potentially improving adherence (4). Extensive literature has shown that dosing schedule can significantly impact adherence and is a major factor that physicians consider in prescribing T2D drugs (5, 6). By the incorporation of dosing schedule, medication efficiency places a direct emphasis on the impact of a medication on the patient’s health and aligns with the patient-centric approach to healthcare. Second, while cost-effectiveness focuses on evaluating treatments from an economic and utility standpoint, medication efficiency approaches treatments from a technological perspective. It offers a detailed assessment of how each dose affects the specific health indicators being targeted. By measuring the improvement achieved per dose, healthcare providers can tailor treatment plans to achieve optimal health outcomes with minimal medication use. This approach promotes both effectiveness and efficiency in healthcare delivery. Additionally, medication efficiency considers the practicality of treatment plans. Highly efficient medications may encourage better patient adherence, ultimately leading to improved long-term health outcomes.

This study employed the data envelopment analysis (DEA) approach to evaluate branded T2D drugs’ medication efficiencies. DEA is a nonparametric benchmarking method that can take multiple factors into consideration simultaneously to gauge the efficiency of transforming inputs into outputs. We benchmarked T2D drugs using clinical data on its benefits and harms extracted from FDA Online Label Repository and recent studies published on flagship medicine journals. The capability of DEA to assess a variety of factors simultaneously is an appealing feature aligned with the holistic assessment approach promoted by governing bodies. For example, the US FDA has transitioned from a blood glucose (sugar) focused assessment approach to a holistic approach, in which co-benefits and harms of the medicine should also be considered (7). Moreover, the relationship between the dosing frequency, therapeutic benefits and side effects of T2D drugs is complex. DEA is especially suitable for this situation, since as a nonparametric approach it does not require a specific functional form for the relationship between inputs and outputs.

In 2012, the European Association for the Study of Diabetes (EASD) and the American Diabetes Association (ADA) jointly issued consensus guidance outlining a decision cycle for the patient-centered management of T2D (8). This approach considered not only key patient characteristics such as age, weight, cardiovascular disease (CVD), and renal history but also specific factors like the HbA1c lowering effect, hypoglycemic risk, impact on weight, side effects, complexity, costs, and cardiorenal effects. These guidelines incorporated these diverse factors to make recommendations regarding the choice of treatment and advocate for a shared decision-making strategy to formulate a comprehensive management plan. This plan signified a departure from a purely glucose-centric approach to a more holistic one, with a preference for a certain mode of application.

The paper proceeds as follows. Section 2 analyzes the literature. Section 3 describes the methodology, including data and variables. Section 4 shows the results. Section 5 concludes with limitations and future research directions.

## 2 Literature review

This section reviews the existing literature on concepts related to medication efficiency (e.g., efficacy and cost-effectiveness), development of holistic assessment of T2D interventions, and the application of benchmarking methods in healthcare studies.

A large body of research, based on both clinical trials and meta-analysis, has evaluated the effectiveness of various diabetes medications, including insulin therapies, metformin, sulfonylureas, GLP-1 receptor agonists, SGLT2 inhibitors, DPP-4 inhibitors, and thiazolidinediones (9). The central function of the diabetes drugs is their capability of controlling the level of blood sugar (glucose), usually measured by the hemoglobin A1c (HbA1c) level (10). Other factors, particularly weight loss and cardiovascular protection, are also important and have been examined through many clinical trials (11–13). Weight loss is a desirable property, since obesity is a major cause of T2D and lowering weight is beneficiary for glucose control (14).

A substantial volume of effectiveness analyses on T2D treatments focused on their cost-effectiveness or cost-utility (15–17). Cost-effectiveness, as a concept in health economics, is typically measured as monetary cost/saving per life years gained or quality-adjusted life years (QALYs) gained. Costs can be evaluated from different perspectives, including health care system, society, and patient. QALY, as a widely used health outcome variable, is intended to combine the length of life with quality of life into a single numerical value. For example, a study (15) identified cost-saving and very cost-effective T2D interventions. However, QALY, the central element in most cost-effectiveness studies, has been constantly criticized for its methodological, ethical and contextual limitations (18).

Furthermore, recent literature shows a trend of holistic assessment of T2D interventions. While early effectiveness assessment of diabetes drugs focuses on HbA1c, the arrival of new evidence indicates that the drugs’ benefits and risks in other aspects should be taken into account (13). As noted in Wilcox et al. (7), prior to 2008, the approval of new antidiabetic drugs by the U.S. FDA primarily depends on their ability to lower blood glucose levels. Since late 2008, the U.S. FDA started to mandate cardiovascular outcome trials (CVOTs) for cardiovascular safety of new antidiabetic agents to ensure their cardiovascular safety. The introduction of CVOTs has fundamentally changed clinical practice guidelines for managing T2D. Moreover, new clinical trial results are constantly popping out and revising the existing knowledge on T2D drugs. For example, recent studies show that GLP-1 drugs offer a slew of benefits including kidney protection and alleviation of depression (19, 20), but are also associated with higher risks of pancreatitis, gastroparesis, and bowel obstruction (21). These benefits and adverse effects, gradually realized through costly clinical trials over a long-time horizon, should be considered in T2D management. Consequently, medical research has increasingly call for holistic assessment of diabetes drugs (7).

The necessity of holistic assessment prompts us to choose the directional distance function method. The directional distance function approach, employed in our study, belongs to the broad class of nonparametric efficiency benchmarking models (22). The benchmarking models, particularly DEA, have been extensively used in healthcare research, to evaluate the efficiency of healthcare systems, hospitals, dialysis facilities and other entities (23–29). More pertinent to our study, an emerging stream of literature started to use DEA to assess medical treatments and diagnosis. A study (30) ranked a set of surgical services (e.g., cardiovascular and plastic surgeries) in a specific hospital through DEA, treating bed turnover, number of physicians, bed occupancy rate as inputs, and numbers of operations and discharged patients as outputs. Another research (31) applied DEA to evaluate the efficiency of hip fracture surgeries, with inputs capturing the pre-surgery conditions of patents and outputs capturing the post-surgery outcomes. A recent work (32) used DEA to assess the efficiency of magnetic resonance imaging. An early research (33) evaluated the efficiency of physical therapy after total knee replacement surgery at the patient level by DEA. We extended this stream of literature to the benchmarking of T2D medications.

Traditional nonparametric benchmarking models suffers from two limitations. First, all variables should assume positive values for the models to be well defined and logically coherent (34). Various new models have been introduced to expand the application of DEA to negative data, including the range directional distance function approach (35), the semi-oriented radial measure approach (36), and the modified slacks-based measure approach (37), among others. Since the attributes of diabetes drugs, such as the change of weight, could be negative, we used the range directional distance function approach. Another limitation is about the undesirable outputs (38), the presence of which breaks the assumption of traditional models that more outputs are more desirable. Different models have been proposed to address undesirable outputs (39–41). There is no clear-cut answer as to which model should be used since each model has its strengths and weaknesses (38).

The above literature survey reveals two significant research gaps that our study seeks to address. First, while there is a considerable body of research assessing the efficiency of treatments for Type 2 Diabetes (T2D), these studies concentrate on cost efficiency. This focus overlooks critical factors such as the dosing schedule and the overall convenience for patients. Second, most benchmarking studies in this field tend to be conducted at the organizational level, evaluating the performance of healthcare providers or facilities rather than focusing on individual medications. There is a need for more granular analyses that evaluate the efficiency of individual treatments.

## 3 Materials and methods

### 3.1 Variables and data

To evaluate the benefits and adverse effects of T2D treatments relative to dosing schedule, we employed *dose frequency* as the single input. Medication frequency referred to how often a person takes a prescribed drug, and it was an important factor in ensuring the effectiveness and safety of the treatment. Lower dose frequency was desirable, since it had been shown to be associated with improvement in patient adherence, patient quality of life, patient satisfaction, and costs (42).

We considered two desirable outputs, change of HbA1c and change of weight, the latter of which may assume negative values. HbA1c was the main biomarker used to assess the average blood sugar level for the past 2 to 3 months. We denoted reduction of HbA1c as positive so decline of HbA1c was desirable. For change of weight, since some drugs might cause undesirable weight gain, we denoted reduction as positive value and increment as negative value. We considered one undesirable output, the all-cause mortality. All-cause mortality was given as the odds ratio between the drug and the placebo, with lower values corresponding to lower mortality. The value of all-cause mortality was always positive.

We obtained T2D medication data from two sources, FDA Online Label Repository^1^ and recent meta-analyses published on flagship medicine journals. FDA Online Label Repository contains the most recent drug listing information that companies have submitted to FDA. Each label featured a *Clinical Studies* section where the companies reported clinical trials data including change in HbA1c and weight against placebos. We obtained the list of T2D drugs from a University of California at San Francisco website.^2^ For each drug in the list, we searched its brand name in FDA Online Label Repository and extracted the clinical trial data, including dose frequency, HbA1c change and weight change, as well as the year when the drug was approved.

As an example, Onglyza is the drug brand name for compound saxagliptin as a DPP4 inhibitor. According to its FDA label,^3^ Onglyza was approved by FDA in 2009. The “recommended dosage of ONGLYZA is 2.5 mg or 5 mg once daily,” so its dose frequency was 1/day. The label may report clinical trial results for the drug as monotherapy or in combination with other drugs. We focus the results of monotherapy. Onglyza’s label indicates that “ONGLYZA was not associated with significant changes from baseline in body weight or fasting serum lipids compared to placebo.” Therefore, the weight change was set to zero. The label reports the “Glycemic Parameters at Week 24 in a Placebo-Controlled Study of ONGLYZA Monotherapy” and indicates a −0.6 difference from placebo with a *p*-value less than 0.0001. Therefore, Onglyza’s HbA1c reduction is 0.6. It is notable that companies may design and carry out the clinical trials for different drugs in different ways. For example, trials for different drugs may have different sample sizes and durations. We use the data as reported in the studies.

The data extracted from the FDA Online Label Repository allowed us to undertake branded drug-level analysis. However, the FDA labels did not report sufficient information on cardiovascular protection, which was recognized as a critical factor in diabetes treatment recently. For the purpose of all-around assessment, we resorted to meta-analysis for further information. We obtained data for compound-level analysis from meta-analysis published on flagship medical journals. We extracted the HbA1c and mortality data from Tsapas et al. (13). The paper reported the effectiveness of 20 glucose-lowering drugs for T2D through meta-analysis. The paper did not report weight change resulted from the interventions, another critical outcome factor affecting quality of life and closely associated with other diseases. Therefore, we complemented the weight change data from other studies (11, 43).

Table 1 showed the summary statistics of input and output variables. We noted that several classes of drugs, such as Biguanides, Glinides, Thiazolidinediones, and Dopamine D2 receptors, contained negative values in weight change, indicating that they could cause weight gain.

**Table 1:** Summary statistics of the variables.

Class	Sulfonylureas	Biguanides	Glinides	Thiazolidinediones	GLP-1 analogs	SGLT2 inhibitors	DPP-4 inhibitors	Dopamine D2 receptors	Combination oral pills
Dose frequency (per day)									
Mean	1.000	1.400	2.500	1.000	0.651	1.000	1.000	1.000	1.462
S.D.	–	0.300	0.500	–	0.518	0.000	0.000	–	0.269
Median	1.000	1.000	2.500	1.000	0.140	1.000	1.000	1.000	1.000
Min	1.000	1.000	2.000	1.000	0.140	1.000	1.000	1.000	1.000
Max	1.000	2.000	3.000	1.000	2.000	1.000	1.000	1.000	2.000
HbA1c change (%)									
Mean	1.700	1.068	0.450	0.800	0.884	0.642	0.667	0.400	0.884
S.D.	–	0.452	0.005	–	0.098	0.044	0.013	–	0.170
Median	1.700	0.700	0.450	0.800	0.800	0.600	0.600	0.400	0.700
Min	1.700	0.500	0.400	0.800	0.500	0.400	0.600	0.400	0.400
Max	1.700	1.800	0.500	0.800	1.300	0.910	0.800	0.400	1.600
Weight change (%)									
Mean	0.000	0.000	−2.815	−2.200	2.344	2.338	0.000	−0.340	0.489
S.D.	–	0.304	0.470	–	5.378	0.463	0.000	–	1.943
Median	0.000	−0.250	−2.815	−2.200	1.430	2.570	0.000	−0.340	0.000
Min	0.000	−0.470	−3.300	−2.200	0.740	1.510	0.000	−0.340	−2.380
Max	0.000	0.640	−2.330	−2.200	7.340	3.080	0.000	−0.340	2.790
Branded drugs	Glucotrol XL	Glucophage, Glumetza, Riomet, Glucophage XR, Fortamet	Prandin, Starlix	Avandia	Victoza, Bydureon, Trulicity, Adlyxin, Ozempic, Mounjaro, Byetta	Farxiga, Jardiance, Steglatro, Brenzavvy, Invokana	Januvia, Onglyza, Nesina	Cycloset	Glucovance, Janumet, PrandiMet, Kombiglyze XR, Jentadueto, Janumet XR, Oseni, Invokamet, Xigduo XR, Glyxambi, Synjardy, Trijardy XR, Kazano

### 3.2 Data envelopment analysis

**Figure 2:**
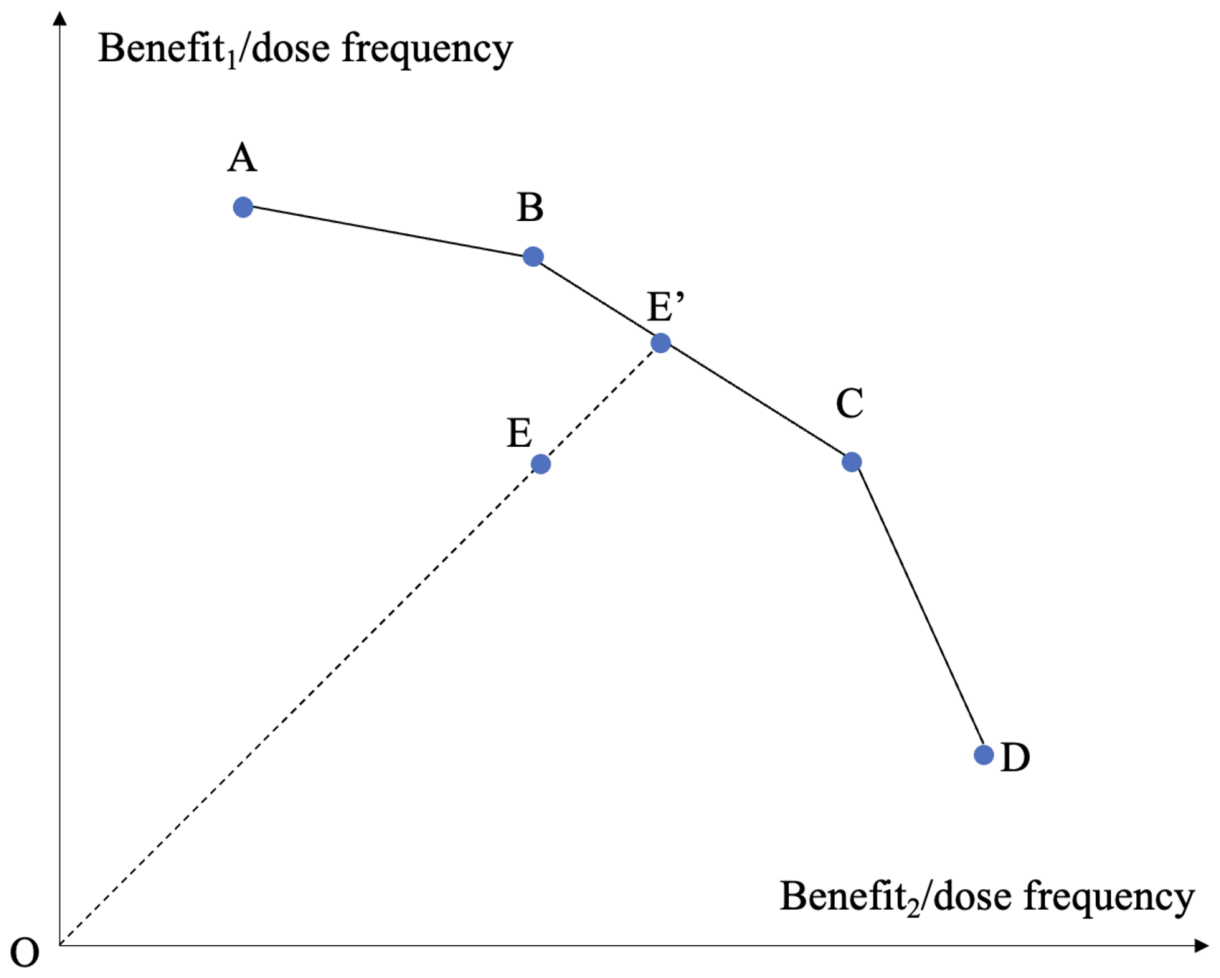
Conceptual depiction of the classical DEA model under variable returns-to-scale.

The assessment of the medication efficiency of T2D drugs was a multidimensional problem by nature, because the treatments involve multiple benefits and adverse effects. We employed DEA to address the multiple factors involved. DEA, as a non-parametric method employing linear programming techniques, identifies an efficiency frontier and measures the distance of each unit from this frontier (22). The primary benefit of DEA in this analysis was its ability to integrate multiple inputs and outputs, even when they were measured in varied units, aligning with the holistic nature of drug assessment. Figure 2 depicts the concept of classical DEA model in the case of evaluating the treatments based on two types of benefits under variable returns-to-scale (VRS) (44). In the figure, there are five treatments labeled A to E. Each treatment is characterized by its dose frequency and two benefits, with the ratios between benefits and dose frequency corresponding to the two axes. The higher the ratio, the more desirable the treatment is. The piecewise linear curve A-B-C-D forms a frontier. The curve is termed a frontier because it is impossible to find two points on it such that one point dominates the other in both ratios. Treatment E is enclosed by the frontier A-B-C-D and thereby is deemed inefficient. Its level of inefficiency is represented by its distance to the frontier, on which we can find a point E’ that dominates E. If we use radial measure for distance, the efficiency of E is OE/OE’. Points B and C serve as the references of E since E’ is derived from the linear combination of B and C. The two-dimensional case can be extended to high-dimensional situation.

A variety of DEA models have been developed. To benchmark the medication efficiency of diabetes drugs, we needed an approach that could (I) integrate multiple factors into account to yield a holistic measure to align with the recent trends, (II) handle negative data, and (III1) handle undesirable outputs. Due to the requirements, we employed the range directional model (RDM) to evaluate the medication efficiency of diabetes drugs (35). While various models have been developed to address negative data, RDM remains the most widely used one.

We used the following notations. Suppose there are n different drugs to be benchmarked, denoted by i=1,…,n . The drug i is characterized by *m* types of inputs, labeled as $x_{ij}$ with j=1,…,m . Treating with drug i generate *s* desirable outcomes labeled as $y_{ir}$ with r=1,…,s , and h undesirable outcomes labeled as $z_{\mathrm{if}}$ with f=1,…,h . Note that $x_{ij}\in {\mathbb{R}}_{+}$ so all inputs are strictly positive, whereas $y_{ir}\in \mathbb{R}$ and $z_{\mathrm{if}}\in \mathbb{R}$ so outputs can assume negative values. Let $\lambda_{i}$ be the weight assigned to the drug i .

Let o denote the drug to be evaluated. For drug o , RDM defines the range of possible improvement for its inputs and desirable outputs as,


(1a) $$R_{oj}^{x}=x_{oj}-\min_{i}\left\{x_{ij}\right\}\text{,}\mathrm{for}j=1,\;\ldots ,\;m;\;\left(1a\right)\text{;}$$

(1b) $$R_{\mathrm{or}}^{y}=\max_{i}\left\{y_{ir}\right\}-y_{\mathrm{or}}\text{,}\mathrm{for}r=1,\ldots ,s\text{.}$$

As the name implies, the ranges of possible improvement in (1a) and (1b) are the maximum possible contraction the input can achieve and the maximum possible expansion the output can achieve. Even with negative data, (1a) and (1b) cannot be negative. For the undesirable output $z_{\mathrm{if}}$ , we take the negative of it and treat $-z_{\mathrm{if}}$ as the desirable output,

for


(1c) $$R_{\mathrm{of}}^{z}=\max_{i}\left\{-z_{\mathrm{if}}\right\}+z_{\mathrm{of}}=z_{\mathrm{of}}-\min_{i}\left\{z_{\mathrm{if}}\right\}\text{,}f=1,\ldots ,h\text{.}$$

Note that in $\left(1c\right)$ , $R_{\mathrm{of}}^{z}$ is similar to $R_{oj}^{x}$ in functional form, implying that the range of possible improvement for undesirable output z is obtained in the same manner as the input x . This treatment of undesirable outputs as inputs is essentially in the spirit of Hailu and Veeman (39).

RDM in its general form is formulated as the following linear program,


$$\theta^{*}=\max\;\theta$$

$$\mathrm{subject to}:\overset{n}{\underset{i=1}{\sum }}\lambda_{i}x_{ij}+\theta R_{oj}^{x}\leq x_{oj},j=1,\ldots ,m\text{;}$$

(2) $$\overset{n}{\underset{i=1}{\sum }}\lambda_{i}y_{ir}-\theta R_{\mathrm{or}}^{y}\geq y_{\mathrm{or}},r=1,\ldots ,s\text{;}$$

$$\overset{n}{\underset{i=1}{\sum }}\lambda_{i}z_{\mathrm{if}}+\theta R_{\mathrm{of}}^{z}\leq z_{\mathrm{of}},f=1,\ldots ,h\text{;}$$

$$\overset{n}{\underset{i=1}{\sum }}\lambda_{i}=1\text{;}$$

$$\lambda_{i}\geq 0,i=1,\ldots ,n\text{.}$$

The above linear program contains n+1 decision variables: θ and ${\left\{\lambda_{i}\right\}}_{i=1,\ldots ,n}$ . Note that above model assumes VRS. It can be proved that the above RDM has the properties of translational invariance and unit invariance (35). Both properties are desirable as they contribute to the comparability and flexibility of the analysis. They ensure that efficiency scores are not distorted by changes in scale or the choice of measurement units, allowing for meaningful and consistent evaluations of the relative efficiency of different units. RDM as in (2) is given as a general form and is non-oriented. It seeks to simultaneously contract the inputs via $\theta R_{oj}^{x}$ , expand the desirable outputs via $\theta R_{\mathrm{or}}^{y}$ and reduce the undesirable outputs via $\theta R_{\mathrm{of}}^{z}$ .

In (2), ${\left\{\lambda_{i}\right\}}_{i=1,\ldots ,n}$ forms a vector of weights to construct the efficiency frontier as a linear combination of all observed inputs and outputs of all drugs. A larger θ implies that the drug under evaluation characterized by $\left(\left\{x_{oj}\right\},\left\{y_{\mathrm{or}}\right\},\left\{z_{\mathrm{of}}\right\}\right)$ , is further away from the efficiency frontier. Therefore, model (2) yields an inefficiency score $\theta^{*}$ , bounded between 0 and 1. The efficiency is obtained as $1-\theta^{*}$ . If efficiency equals one, the drug is on the frontier and attains a perfect efficiency score. Model (2) is solved once for each of the n drugs. So collectively we solved the optimization problem as described in (2) n times.

Further exposition on the choice of DEA for benchmarking in this study is needed. Another popular benchmarking method is stochastic frontier analysis (SFA). For the purpose of this study, DEA enjoys several advantages over SFA. As a nonparametric method, DEA does not require a specific functional form for the relationship between inputs and outputs. This flexibility is beneficial since the exact nature of the relationship between dosing frequency and the outputs is complex. Additionally, DEA is well-suited to handle multiple outputs, such as glucose-lowering capability (a desirable output) and side effects (an undesirable output). SFA, on the other hand, typically handles a single output, making it less appropriate when dealing with the multidimensional nature of medication outcomes. Furthermore, DEA can easily incorporate undesirable outputs into the analysis using models like the directional distance function. This is more straightforward in DEA than in SFA, where handling undesirable outputs would require more complex modeling adjustments. Last but not least, DEA can perform well even with smaller sample sizes, as in our study of 20 T2D drugs. SFA typically requires larger sample sizes to provide reliable estimates, as it depends on statistical noise and parameter estimation.

All data analyses and computations were carried out using R version 4.2.3 (R Project for Statistical Computing), during November 2023 and February 2024.

## 4 Results

Table 2 reported the medication efficiencies of 38 T2D branded drugs, ranked from high to low. The results indicated that there were five medications that achieve the highest efficiency of unity, including Glucotrol XL, Glucophage, Riomet, Ozempic, and Mounjaro. Among them, Ozempic and Mounjaro belong to the GLP-1 class of drugs, a new generation of drugs recently approved by FDA to treat T2D, and have been praised for their weight-loss benefits. The results in Table 2 did not reflect the transition to holistic assessment since 2008, as it did not include cardiovascular and other factors that had been shown to be associated with the drugs and affected the mortality rate of the patients. We undertook further analysis based on Model (2) to incorporate mortality rate as undesirable output, using data from meta-analysis published on flagship medicine journals.

**Table 2:** Medication efficiency of T2D branded drugs.

Rank	Branded drug	Approval year	Efficiency	Rank	Branded drug	Approval year	Efficiency
1	Glucotrol XL	1994	1	20	Glumetza	1995	0.384
1	Glucophage	1995	1	21	Synjardy	2015	0.380
1	Riomet	1995	1	22	Farxiga	2014	0.376
1	Ozempic	2017	1	23	Kombiglyze XR	2010	0.375
1	Mounjaro	2022	1	24	Adlyxin	2016	0.374
6	Janumet XR	2012	0.857	25	Janumet	2007	0.361
7	Jentadueto	2012	0.564	26	Onglyza	2009	0.353
7	Kazano	2013	0.564	26	Nesina	2013	0.353
9	Glucovance	2000	0.553	28	Steglatro	2017	0.351
10	Oseni	2013	0.545	29	Prandin	1997	0.351
11	Victoza	2010	0.534	30	Fortamet	2004	0.347
12	Trulicity	2014	0.524	31	Brenzavvy	2023	0.346
13	Invokana	2013	0.485	32	Xigduo XR	2014	0.343
14	Bydureon	2012	0.463	33	Byetta	2005	0.342
15	PrandiMet	2008	0.463	34	Glucophage XR	2000	0.332
16	Jardiance	2014	0.456	35	Glyxambi	2015	0.316
17	Januvia	2006	0.400	35	Trijardy XR	2020	0.316
18	Invokamet	2014	0.392	37	Cycloset	2009	0.314
19	Avandia	1999	0.387	38	Starlix	2000	0.294

The computation was based on the RDM approach in (2) and employs dose frequency as input, HbA1c decrease and weight loss as outputs. The data for inputs and outputs were from FDA Online Label Repository.

Since meta-analysis reported results for the underlying compound rather the branded drugs, our further assessment was conducted at the compound-level. Figure 2 plotted the evolution of medication efficiency of T2D drugs. It showed that the new generation of T2D drugs in the GLP-1 class including oral Semaglutide, subcutaneous Semaglutide and Dulaglutide, all of which were approved in recent years, attained the perfect efficiency of one. Lixisenatide, as another GLP-1 agent, also achieved a high efficiency of 0.85. Exenatide, as the first GLP-1 agent approved by FDA, had a low medication efficiency of 0.15. But the ensuing extended-release Exenatide, approved in 2012, had significantly improved and attained an efficiency of 0.47. Liraglutide, an earlier GLP-1 drug, had lower efficiency of 0.24. Earlier T2D drugs, such as Sulphonylureas and premixed insulin, had lower medication efficiencies.

**Figure 3:**
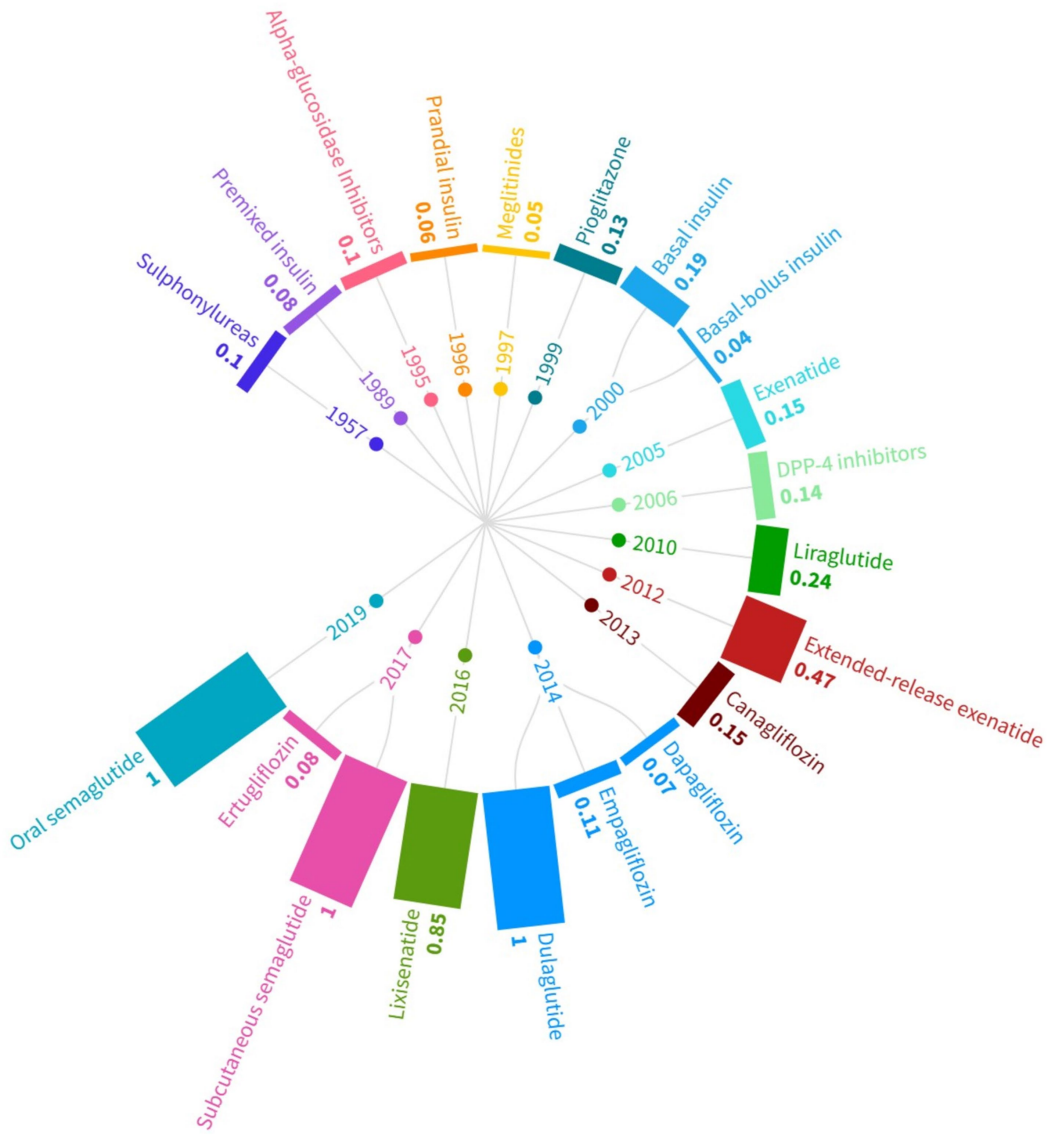
Evolution of medication efficiency of T2D drugs. The computation was based on the RDM approach in (2) and employed dose frequency as input, HbA1c decrease and weight loss as desirable outputs, mortality rate as undesirable output. The year was the first approval year by FDA. The data for inputs and outputs were from meta-analysis published on flagship medical journals.

Figure 3 clearly indicated the technological progress of T2D drugs over the past decades. None of the drugs approved before 2010 was able to achieve a medication efficiency greater than 0.2. The first drug that broke the 0.2 efficiency threshold was Liraglutide, approved in 2010 as an GLP-1 class drug. The average medication efficiency of pre-2010 drugs was merely 0.104, whereas post-2010 (including 2010) drugs had an average efficiency of 0.498, almost five times of the pre-2010 level.

**Figure 4:**
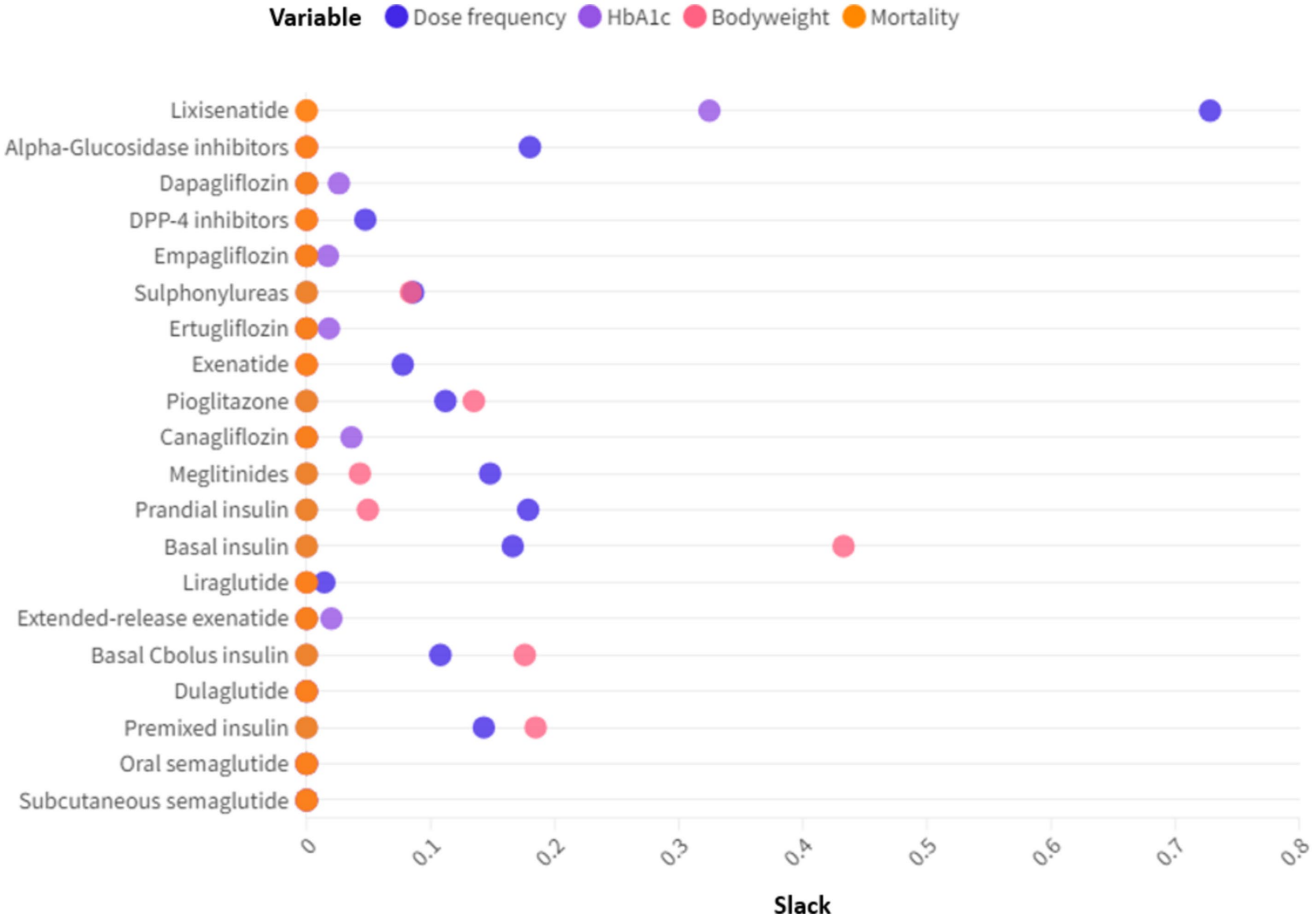
Slack analysis of the T2D drugs. Slacks refer to the differences between the actual inputs/outputs and the ideal inputs/outputs.

DEA can provide not only the efficiency levels of the drugs but also slack values. Slacks refer to the shortfall of a drug from its efficient counterpart on the frontier. In the context of production economics, knowing the slacks can help the inefficient unit to identify improvement opportunities. While for drugs improving the inputs/outputs is not as viable as a manufacturing plant, it is still valuable to know the slacks since they give rise to efficiency gaps. We undertook slack analysis to identify the shortfalls of the drugs from the efficient frontier. Figure 4 plotted the slacks for the 20 T2D drugs across the input and outputs. Larger slack values indicated that the variable was further away from the frontier. For drugs on the frontier, all slacks were zero. Figure 4 showed that for 8 of the 20 drugs, the biggest slack occurred for its dose frequency. HbA1c reduction and bodyweight decline accounted for 5 and 4 of the biggest slacks, respectively. Therefore, high dose frequency, low HbA1c reduction and marginal bodyweight decline were common causes of inefficiencies.

## 5 Discussion and conclusion

The paper benchmarked the medication efficiency of T2D drugs and their technological progress, based on DEA approach and clinical data. The concept of medication efficiency refers to the blood sugar control capability, weight-loss effect and mortality rate, relative to the dosing frequency. Namely, it measures how much benefit and harm can be caused per dose. Medication efficiency offers an alternative perspective on T2D drugs and complements existing measures such as cost-effectiveness. Due to the existence of negative values and undesirable output in benefits and adverse effects, we used a special DEA model, the range directional model, to compute the efficiency of the drugs. We found that some newer generation GLP-1 drugs (oral Semaglutide, subcutaneous Semaglutide, Dulaglutide) exhibited significantly higher efficiencies than other drugs. Moreover, earlier medications (e.g., Sulphonylureas, Premixed insulin, *α*-glucosidase inhibitors) had medication efficiencies less than 1. The inefficiencies were caused by gaps in dosing frequency, Hb1Ac reduction and bodyweight decline.

A potential application of the benchmarking results is the development of combination therapies for T2D by identifying points on the frontier that may dominate inefficient treatments. As illustrated in Figure 2, DEA method constructs a piecewise linear frontier through linear combination of sample points and measures the efficiency by comparing an observed point against a hypothetical point on the frontier. A critical problem in this benchmarking process is that whether the hypothetical and superior point, as a linear combination of sample points, could be realized in reality. If the drugs had linear dose–response and the responses of different drugs were additive, the hypothetical point would be realizable. For example, DEA results showed that Canagliflozin was benchmarked against a linear combination of subcutaneous Semaglutide and Dulaglutide with weights 0.783 and 0.217, respectively. If there is a linear association between dose and response for subcutaneous Semaglutide and Dulaglutide and the responses from the two drugs are additive, then combining subcutaneous Semaglutide and Dulaglutide according to their weights in benchmarking could generate favorable effects. Determining the dose–response relationship for a drug is a demanding and costly task. There was evidence that dose and weight loss are linearly associated for Semaglutide (45).

There are several limitations of this study. T2D drugs can exhibit a wide range of adverse side effects, including but not limited to urinary tract infections, fractures, amputations (46). While we have included mortality rate as an aggregate measure of adverse events, most side effects are not life-threatening. Another concern is that the research hinges on clinical trials data. Even though we have restricted our sample to FDA documents and meta-analysis on flagship academic journals to safeguard data accuracy, it is common in medical research that new results from more robust trials may amend earlier findings. Therefore, emergence of new clinical results may override some of the results in this study. Finally, the choice of treatment for T2D is very complex. It depends on not only the drugs’ attributes but also the patients’ characteristics such as age, weight and medical history (7). The analysis in this paper aimed to shed light on medication efficiency and technological progress. It did not constitute recommendations for choice of treatment.

## Data Availability

The raw data supporting the conclusions of this article will be made available by the authors, without undue reservation.

## Footnotes

^1^https://labels.fda.gov/, accessed on November 29th, 2023.

^2^https://dtc.ucsf.edu/types-of-diabetes/type2/treatment-of-type-2-diabetes/medications-and-therapies/type-2-non-insulin-therapies/table-of-medications/, accessed on November 29th, 2023.

^3^https://www.accessdata.fda.gov/drugsatfda_docs/label/2015/022350s016lbl.pdf, accessed on November 29th, 2023.
